# A novel oncogenic pathway by TLS–CHOP involving repression of MDA-7/IL-24 expression

**DOI:** 10.1038/bjc.2012.199

**Published:** 2012-05-15

**Authors:** K Oikawa, M Tanaka, S Itoh, M Takanashi, T Ozaki, Y Muragaki, M Kuroda

**Affiliations:** 1 Department of Molecular Pathology, Tokyo Medical University, 6-1-1 Shinjuku, Shinjuku-ku, Tokyo 160-8402, Japan; 2 First Department of Pathology, Wakayama Medical University, 811-1 Kimiidera, Wakayama-shi, Wakayama 641-8509, Japan

**Keywords:** TLS–CHOP, FUS-DDIT3, myxoid liposarcoma, MDA-7/IL-24

## Abstract

**Background:**

Translocated in liposarcoma-CCAAT/enhancer binding protein homologous protein (TLS–CHOP) (also known as FUS-DDIT3) chimeric oncoprotein is found in the majority of human myxoid liposarcoma (MLS), but its molecular function remains unclear.

**Methods:**

We knockdowned TLS–CHOP expression in MLS-derived cell lines by a specific small interfering RNA, and analysed the gene expression profiles with microarray.

**Results:**

TLS-CHOP knockdown inhibited growth of MLS cells, and induced an anticancer cytokine, melanoma differentiation-associated gene 7 (MDA-7)/interleukin-24 (IL-24) expression. However, double knockdown of TLS–CHOP and MDA-7/IL-24 did not inhibit MLS cell growth.

**Conclusion:**

Repression of MDA-7/IL-24 expression by TLS–CHOP is required for MLS tumour growth, and TLS–CHOP may become a promising therapeutic target for MLS treatment.

More than 90% of human myxoid liposarcoma (MLS) cases are associated with the chromosomal translocation, which creates a chimeric oncogene comprising part of the *TLS* (*translocated in liposarcoma*) gene (also known as *FUS* (*fused in Ewing’s sarcoma*)) and part of the *CHOP* (*CCAAT/enhancer binding protein* (*C/EBP*) *homologous protein*) gene (also called *DDIT3* (*DNA damage-inducible transcript 3*) and *GADD153* (*growth arrest- and DNA damage-inducible gene 153*)) (Crozat *et al*, 1993; Rabbitts *et al*, 1993; Powers *et al*, 2010). The resultant fusion gene *TLS–CHOP* encodes the N-terminal half of TLS fused to complete sequence of CHOP (Powers *et al*, 2010; Figure 1A). TLS-CHOP protein is considered to function as an abnormal transcription factor (Kuroda *et al*, 1999; Pérez-Mancera *et al*, 2008; Andersson *et al*, 2010). The definitive TLS–CHOP function for MLS development, however, is unclear.

Melanoma differentiation-associated gene 7 (MDA-7)/interleukin-24 (IL-24) protein is expressed in cells of the immune system and normal human melanocytes (Jiang *et al*, 1995; Wolk *et al*, 2002). Exogenous expression of MDA-7/IL-24 induces growth arrest and apoptotic cell death in various human malignant cells (Dash *et al*, 2010; Rahmani *et al*, 2010).

In this report, we have found a novel pathway of TLS–CHOP with MDA-7/IL-24 repression.

## Materials and methods

### Cell culture

The MLS-derived cell lines, 1955/91 and 2645/94, were kindly provided from Professor David Ron (University of Cambridge), and were cultured in Dulbecco’s modified Eagle’s medium (Sigma-Aldrich Corporation, St Louis, MO, USA) supplemented with 10% foetal bovine serum. Cell quantification was performed as previously described (Oikawa *et al*, 2004).

### Small interfering RNA transfection

Small interfering RNA (siRNA) transfection (1 *μ*ℳ final concentration) was performed as previously described (Oikawa *et al*, 2004). The nucleotide sequences of the chemically synthesised double-stranded siRNAs are as follows: *TLS–CHOP* siRNA, 5′-GGAAGUGUAUCUUCAUACAdTdT-3′ *MDA-7/IL-24* siRNA, 5′-GUGGAUGGGUGCUUAGUAAdTdT-3′ and negative control siRNA, 5′-AUCCGCGCGAUAGUACGUAdTdT-3′.

### Detection of *TLS–CHOP* variants and quantitative real-time PCR analysis

RNA isolation and first-strand cDNA synthesis were performed as previously described (Oikawa *et al*, 2008a). For detection of *TLS–CHOP* variants, we performed PCR analysis with *TLS–CHOP* detection primers 5′-CTTATGGCCAGAGCCAGAAC-3′ and 5′-AAGGCAATGACTCAGCTGCC-3′. The amplification products were sequenced with ABI PRISM 310 Genetic analyser (Applied Biosystems, Foster City, CA, USA). Real-time PCR analysis was performed as previously described (Oikawa *et al*, 2008b) using *TLS–CHOP*-specific primers 5′-ATGAACGGCTCAAGCAGGAA-3′ and 5′-TGGTGCAGATTCACCATTCG-3′, and *MDA-7/IL-24*-specific primers 5′-GTTTTCCATCAGAGACAGTG-3′ and 5′-GTAGAATTTCTGCATCCAGG-3′. The *TLS–CHOP* and *MDA-7/IL-24* mRNA levels were normalised to *β-actin* signals (Oikawa *et al*, 2004). We performed real-time PCR analysis in duplicate.

### Microarray analysis

Cells were transfected with the *TLS–CHOP* or negative control siRNAs, and were incubated for 72 h. Biotin-labelled complementary RNA (cRNA) was then generated from 1 *μ*g of total RNA of the cells using CodeLink iExpress Expression Assay Reagent Kit (GE Healthcare UK Ltd, Buckinghamshire, UK), and was hybridised to CodeLink Human Whole Genome Bioarray (GE Healthcare) using iAmplify cRNA Preparation and Hybridisation Reagents Kit (GE Healthcare) according to Expression Bioarray System User Guide ver. 2.0. The array slides were incubated for 21 h at 37 °C with shaking, and were scanned with a DNA microarray scanner G2505A (Agilent Technologies, Inc., Santa Clara, CA, USA). The scanned images were analysed and median normalised using CodeLink Expression Analysis Version 4.1.0.29054 (GE Healthcare). The data have been deposited in NCBI’s Gene Expression Omnibus and are accessible through GEO Series accession number GSE33616.

### Western blot analysis

Western blot analysis was performed as previously described (Oikawa *et al*, 2002). Anti-TLS–CHOP monoclonal antibody (clone 14) was previously generated (Oikawa *et al*, 2006). Monoclonal anti-*α*-tubulin antibody clone B-5-1-2 (T-5168; Sigma) was purchased.

### Plasmid construction and transfection

To create an MDA-7/IL-24 expression vector, cDNA fragment containing the complete coding region of MDA-7/IL-24 was amplified by PCR using the primers 5′-GCGCGGATCCGAGATGAATTTTCAACAGAG-3′ and 5′-GGCCAAGCTTCCTGGTCTAGACATTCAGAG-3′, and inserted into the mammalian expression vector, pcDNA3.1(−) (Invitrogen, Carlsbad, CA, USA). Plasmid transfection was performed using Lipofectamine 2000 reagent (Invitrogen) and Opti-MEM I Reduced-Serum Medium (Invitrogen).

## Results

### TLS–CHOP knockdown represses cell growth of MLS-derived cell lines

**Figure 1:**
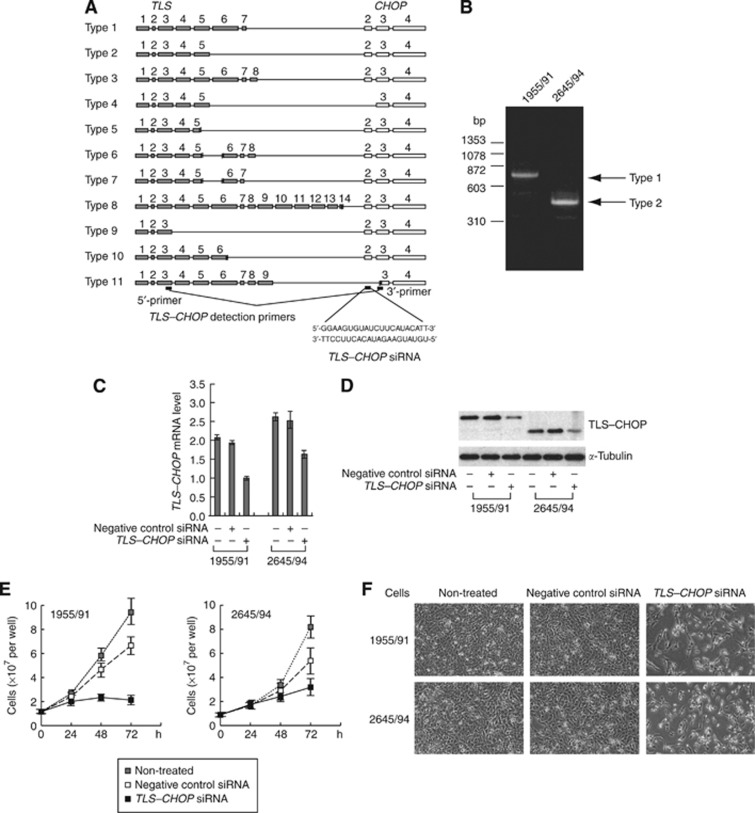
Repression of *TLS–CHOP* expression by *TLS–CHOP* siRNA in MLS-derived cells inhibits cell growth. (**A**) Schematic structures of various types of *TLS–CHOP* fusion gene. Grey and open boxes represent exons of the *TLS* and *CHOP* genes, respectively. The target site of *TLS–CHOP* siRNA and the hybridisation sites of *TLS–CHOP* detection primers are also shown. (**B**) Detection of *TLS–CHOP* transcripts in MLS-derived cell lines. PCR with *TLS–CHOP* detection primers was performed using cDNAs synthesised from total RNAs of MLS-derived cells. The PCR products were fractionated by electrophoresis on a 2% agarose gel. Types of *TLS–CHOP* were determined by direct sequencing of the PCR products. (**C**) Reduction of *TLS–CHOP* transcript in 1955/91 and 2645/94 cells by *TLS–CHOP* siRNA. In all, 72 h after siRNA transfection, total RNA from the cells was extracted and subjected to real-time PCR analysis. Data were normalised to a minimum mRNA level that was arbitrarily set to 1 in the graphical presentation. (**D**) Western blot analysis of total cell extracts from 1955/91 and 2645/94 cells 48 h after siRNA transfection. *α*-Tubulin is shown as a loading control. (**E**) *TLS–CHOP* siRNA inhibits cell growth of MLS-derived cells. 1955/91 and 2645/94 cells were transfected with *TLS–CHOP* siRNA or negative control siRNA. Then, the cells in 12-well culture plates were counted at several time points using a haemocytometer. *Bars*, SD. (**F**) Representative phase-contrast images of 1955/91 and 2645/94 cells at 72 h after siRNA transfection.

First, we examined the activity of the three newly designed effective siRNAs that target different positions of *TLS–CHOP* in a preliminary experiment (Supplementary Figure 1), and selected the most effective siRNA among them (hereafter termed *TLS–CHOP* siRNA) for use in subsequent experiments. The *TLS–CHOP* siRNA targets exon 2 of the *CHOP* gene (Figure 1A). Although types 4 and 11 of *TLS–CHOP* variants do not have the target region, TLS–CHOP in over 80% of MLS is type 1 or 2. We confirmed that the two MLS-derived cell lines, 1955/91 and 2645/94, carries type 1 and type 2, respectively (Figure 1B). TLS-CHOP knockdown by the siRNA inhibited cell growth and induced cell death in both cell lines (Figure 1C–F). On the other hand, a non-targeting negative control siRNA did not affect cell growth, indicating that the effects of *TLS–CHOP* siRNA are not by off-target effects.

### TLS–CHOP knockdown induces MDA-7/IL-24 expression in MLS cells

**Figure 2:**
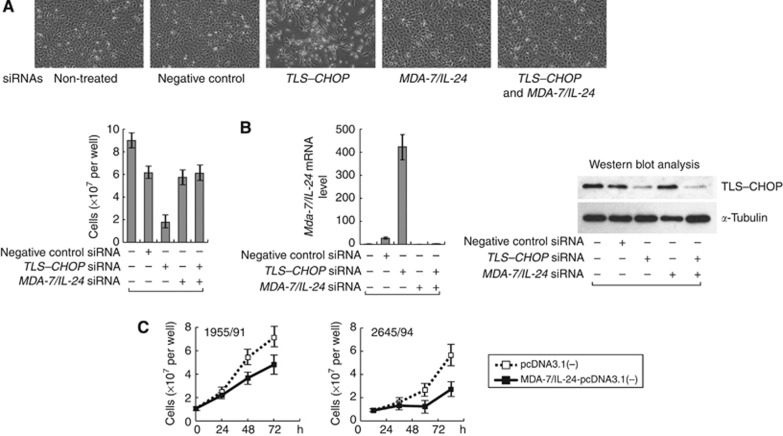
Growth arrest of MLS cells by *TLS–CHOP* siRNA is caused by *MDA-7/IL-24* expression. (**A**) Representative phase-contrast images (upper panels) and cell numbers (lower panel) of 1955/91 cells at 72 h after transfection with *TLS–CHOP* siRNA and/or *MDA-7/IL-24* siRNA, or negative control siRNA. (**B**) Induction of MDA-7/IL-24 expression in 1955/91 cells by *TLS–CHOP* siRNA. In all, 72 h after siRNA transfection, total RNA and protein samples were prepared from the cells and subjected to real-time PCR and western blot analysis, respectively. Left panel shows *MDA-7/IL-24* mRNA level. Data were normalised to the mRNA level of non-treated cells that was arbitrarily set to 1 in the graphical presentation. Right panel shows western blot analysis of TLS–CHOP expression. *α*-Tubulin is shown as a loading control. (**C**) Ectopic expression of MDA-7/IL-24 in MLS cells represses cell growth. 1955/91 and 2645/94 cells were transfected with expression vector. Then, the cells in 12-well culture plates were counted at several time points using a haemocytometer. *Bars*, SD.

Next, we compared mRNA expression profiles of both 1955/91 and 2645/94 cells transfected with *TLS–CHOP* siRNA or negative control siRNA by microarray analysis (see Materials and Methods). We found that several dozen genes showed at least two-fold differential expression by *TLS–CHOP* siRNA (Table 1). Among the genes, we focused on the *MDA-7/IL-24* gene because it encodes an anticancer cytokine (Dash *et al*, 2010). *TLS-CHOP* siRNA induced a significant increase in the expression of *MDA-7/IL-24* in both cell lines (Table 1; Figure 2B). Thus, to confirm that MDA-7/IL-24 is important for growth arrest by TLS–CHOP knockdown, we prepared *MDA-7/IL-24* siRNA and performed double transfection with both *TLS–CHOP* and *MDA-7/IL-24* siRNAs into 1955/91 cells. *MDA-7/IL-24* knockdown cancelled the growth inhibitory effects by *TLS–CHOP* siRNA alone (Figure 2A and B).

### Overexpression of MDA-7/IL-24 represses MLS cell growth

MDA-7/IL-24 displays nearly ubiquitous cancer-specific toxicity (Dash *et al*, 2010; Rahmani *et al*, 2010). To confirm that MDA-7/IL-24 is also toxic for MLS, we transfected 1955/91 and 2645/94 cells with an MDA-7/IL-24 expression vector MDA-7/IL-24-pcDNA3.1(−) or a control vector pcDNA3.1(−). As shown in Figure 2C, MDA-7/IL-24-pcDNA(3.1) transfection represses the growth of the cells.

## Discussion

We have demonstrated that TLS–CHOP knockdown in MLS cells represses cell growth (Figure 1C–E), suggesting that TLS–CHOP plays an essential role for growth of MLS cells. Furthermore, our results suggest that TLS–CHOP may become a promising molecular target for MLS treatment.

TLS-CHOP knockdown in MLS cells induced increased expression of an anticancer cytokine MDA-7/IL-24 (Table 1; Figure 2B). Thus, we consider that although the cancerous characteristics of MLS cells have potential to induce MDA-7/IL-24 expression, TLS–CHOP represses it and contributes to maintain the tumour growth.

In conclusion, we have revealed a novel pathway involving repression of MDA-7/IL-24 expression for tumourigenesis and/or growth of MLS. We believe that our results will contribute understanding of molecular function of the chimeric oncoprotein and development of a novel molecular therapy for cancers.

## Figures and Tables

**Table 1:** Differential expression probes between MLS cells treated with TLS–CHOP and negative control siRNAs

			**Fold change (TLS–CHOP/negative cont.)**	
**Probe name**	**Gene symbol**	**NCBI accession number**	**1955/91**	**2645/94**
GE481001	C14orf34	BF573354.1	2.213	2.860
GE516375	NULL	BC008580.1	11.472	3.080
GE53420	GFPT2	NM_005110.1	4.367	2.468
GE54247	CST7	NM_003650.2	2.708	2.012
GE542691	NRG1	NM_013956.1	8.811	2.171
GE57599	PI3	NM_002638.2	5.223	2.735
GE57919	TNFAIP6	NM_007115.2	4.657	3.037
GE58805	TXNIP	NM_006472.1	4.449	2.253
GE58964	IL-24	NM_181339.1	6.112	2.438
GE59353	MGLL	U67963.1	11.611	2.464
GE59652	CSF3	NM_000759.2	27.532	4.177
GE61078	DHRS2	NM_182908.3	2.835	2.566
GE61301	C3	NM_000064.1	8.036	4.744
GE616218	NULL	BC038580.2	2.768	2.410
GE61968	C9orf26	NM_033439.2	3.057	2.141
GE62312	PTGS2	NM_000963.1	15.187	3.389
GE63376	CXCL1	NM_001511.1	6.019	2.180
GE79458	HAS2	NM_005328.1	17.894	2.772
GE79854	MMP3	NM_002422.2	125.188	4.472
GE79985	LUM	NM_002345.3	6.591	2.425
GE80239	EREG	NM_001432.1	7.313	2.470
GE80932	SOD2	NM_000636.2	5.168	2.864
GE833100	TNC	BQ002165.1	3.189	2.040
GE854770	TMEM46	NM_001007538.1	3.332	2.105
GE86887	HS3ST3B1	BC063301.1	3.554	2.021
GE87032	ZIC2	NM_007129.2	2.360	2.952
GE87518	ECE2	NM_032331.2	2.948	2.319
GE59642	CXCL10	NM_001565.1	0.300	0.226
GE59786	FBLN1	NM_006486.2	0.335	0.445
GE60115	LLGL2	NM_004524.2	0.466	0.261
GE80150	ELF3	NM_004433.3	0.175	0.424
GE892360	NULL	BC050468.2	0.446	0.486

Only the probes showing over twofold change in both two cell lines are listed.

‘NULL’ in Gene symbol column means that the probe sequence is not entried in NCBI database.

## References

[bib1] Andersson MK, Göransson M, Olofsson A, Andersson C, Aman P (2010) Nuclear expression of FLT1 and its ligand PGF in FUS-DDIT3 carrying myxoid liposarcomas suggests the existence of an intracrine signaling loop. BMC Cancer 10: 249 20515481 10.1186/1471-2407-10-249 PMC2889895

[bib2] Crozat A, Åman P, Mandahl N, Ron D (1993) Fusion of CHOP to a novel RNA-binding protein in human myxoid liposarcoma. Nature 363: 640–644 8510758 10.1038/363640a0

[bib3] Dash R, Bhutia SK, Azab B, Su ZZ, Quinn BA, Kegelmen TP, Das SK, Kim K, Lee SG, Park MA, Yacoub A, Rahmani M, Emdad L, Dmitriev IP, Wang XY, Sarkar D, Grant S, Dent P, Curiel DT, Fisher PB (2010) mda-7/IL-24: a unique member of the IL-10 gene family promoting cancer-targeted toxicity. Cytokine Growth Factor Rev 21: 381–391 20926331 10.1016/j.cytogfr.2010.08.004 PMC3164830

[bib4] Jiang H, Lin JJ, Su ZZ, Goldstein NI, Fisher PB (1995) Subtraction hybridization identifies a novel melanoma differentiation associated gene, mda-7, modulated during human melanoma differentiation, growth and progression. Oncogene 11: 2477–2486 8545104

[bib5] Kuroda M, Wang X, Sok J, Yin Y, Chung P, Giannotti JW, Jacobs KA, Fitz LJ, Murtha-Riel P, Turner KJ, Ron D (1999) Induction of a secreted protein by the myxoid liposarcoma oncogene. Proc Natl Acad Sci USA 96: 5025–5030 10220412 10.1073/pnas.96.9.5025 PMC21810

[bib6] Oikawa K, Akiyoshi A, Tanaka M, Takanashi M, Nishi H, Isaka K, Kiseki H, Idei T, Tsukahara Y, Hashimura N, Mukai K, Kuroda M (2008a) Expression of various types of alternatively spliced WAPL transcripts in human cervical epithelia. Gene 423: 57–62 18662753 10.1016/j.gene.2008.07.001

[bib7] Oikawa K, Ishida T, Imamura T, Yoshida K, Takanashi M, Hattori H, Ishikawa A, Fujita K, Yamamoto K, Matsubayashi J, Kuroda M, Mukai K (2006) Generation of the novel monoclonal antibody against TLS/EWS-CHOP chimeric oncoproteins that is applicable to one of the most sensitive assays for myxoid and round cell liposarcomas. Am J Surg Pathol 30: 351–356 16538055 10.1097/01.pas.0000194043.01104.eb

[bib8] Oikawa K, Ohbayashi T, Kiyono T, Nishi H, Isaka K, Umezawa A, Kuroda M, Mukai K (2004) Expression of a novel human gene, human wings apart-like (hWAPL), is associated with cervical carcinogenesis and tumor progression. Cancer Res 64: 3545–3549 15150110 10.1158/0008-5472.CAN-03-3822

[bib9] Oikawa K, Ohbayashi T, Mimura J, Fujii-Kuriyama Y, Teshima S, Rokutan K, Mukai K, Kuroda M (2002) Dioxin stimulates synthesis and secretion of IgE-dependent histamine-releasing factor. Biochem Biophys Res Commun 290: 984–987 11798171 10.1006/bbrc.2001.6302

[bib10] Oikawa K, Yoshida K, Takanashi M, Tanabe H, Kiyuna T, Ogura M, Saito A, Umezawa A, Kuroda M (2008b) Dioxin interferes in chromosomal positioning through the aryl hydrocarbon receptor. Biochem Biophys Res Commun 374: 361–364 18640100 10.1016/j.bbrc.2008.07.044

[bib11] Pérez-Mancera PA, Bermejo-Rodríguez C, Sánchez-Martín M, Abollo-Jiménez F, Pintado B, Sánchez-García I (2008) FUS-DDIT3 prevents the development of adipocytic precursors in liposarcoma by repressing PPARgamma and C/EBPalpha and activating eIF4E. PLoS One 3: e2569 18596980 10.1371/journal.pone.0002569 PMC2434200

[bib12] Powers MP, Wang WL, Hernandez VS, Patel KS, Lev DC, Lazar AJ, López-Terrada DH (2010) Detection of myxoid liposarcoma-associated FUS-DDIT3 rearrangement variants including a newly identified breakpoint using an optimized RT-PCR assay. Mod Pathol 23: 1307–1315 20581806 10.1038/modpathol.2010.118

[bib13] Rabbitts TH, Forster A, Larson R, Nathan P (1993) Fusion of the dominant negative transcription regulator CHOP with a novel gene FUS by translocation t(12;16) in malignant liposarcoma. Nat Genet 4: 175–180 7503811 10.1038/ng0693-175

[bib14] Rahmani M, Mayo M, Dash R, Sokhi UK, Dmitriev IP, Sarkar D, Dent P, Curiel DT, FIsher PB, Grant S (2010) Melanoma differentiation associated gene-7/interleukin-24 potently induces apoptosis in human myeloid leukemia cells through a process regulated by endoplasmic reticulum stress. Mol Pharmacol 78: 1096–1104 20858700 10.1124/mol.110.068007 PMC2993469

[bib15] Wolk K, Kunz S, Asadullah K, Sabat R (2002) Cutting edge: immune cells as sources and targets of the IL-10 family members? J Immunol 168: 5397–5402 12023331 10.4049/jimmunol.168.11.5397

