# Supplementary material for: A novel oncogenic pathway by TLS–CHOP involving repression of MDA-7/IL-24 expression
Source: Br J Cancer. 2012 May 15;106(12):1976–9. doi: 10.1038/bjc.2012.199 (PMC3388565; doi:10.1038/bjc.2012.199)
Supplement: Supplementary Figure 1 [file bjc2012199x1.pdf]

Supplementary Figure 1

A

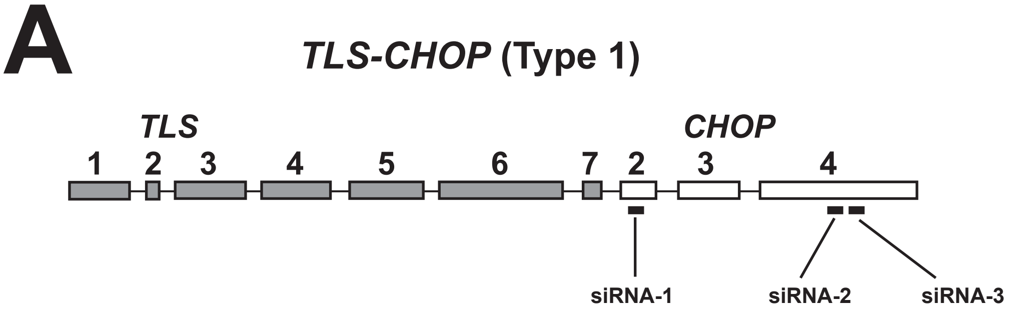

siRNAs specific for *TLS-CHOP*

siRNA-1: GGAAGUGUAUCUUCAUACAdTdT

siRNA-2: AGUGGCACAGCUAGCUGAAdTdT

siRNA-3: UGGUGAAUCUGCACCAAGCdTdT

B

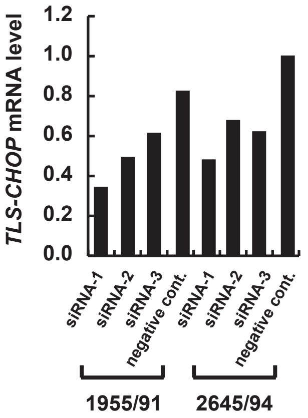

Supplementary Figure 1. The newly-designed siRNAs targeting *TLS-CHOP*. (**A**) The target sites and the nucleotide sequences of the siRNAs. Schematic structure of *TLS-CHOP* fusion gene type 1 is also shown. Gray and open boxes represent exons of the *TLS* and *CHOP* genes, respectively. (**B**) Reduction of *TLS-CHOP* transcript in the myxoid liposarcoma-derived cell lines, 1955/91 and 2645/94 cells, by the siRNAs (siRNA-1, 2, and 3, respectively). 48 h after siRNA transfection, total RNA from the cells was extracted and subjected to real-time PCR analysis. Data were normalized to a maximum mRNA level that was arbitrarily set to 1 in the graphical presentation. Because siRNA-1 (hereafter termed *TLS-CHOP* siRNA) was the most effective at reducing *TLS-CHOP* mRNA levels, we used it in subsequent experiments. Effects of the other two siRNAs on MLS cells were weaker but similar to that of *TLS-CHOP* siRNA (Figures 1 and 2, and data not shown), indicating that the effects were surely caused by TLS-CHOP knockdown.
